# Characterization of circulating extracellular traps and immune responses to citrullinated LL37 in psoriasis

**DOI:** 10.3389/fimmu.2023.1247592

**Published:** 2023-12-19

**Authors:** María Teresa Martín Monreal, Amanda Kvist-Hansen, Laura Massarenti, Rudi Steffensen, Nikolai Loft, Peter Riis Hansen, Niels Ødum, Lone Skov, Claus H. Nielsen

**Affiliations:** ^1^ Institute for Inflammation Research, Center for Rheumatology and Spine Diseases, Rigshospitalet, Copenhagen University Hospital, Copenhagen, Denmark; ^2^ Department of Demartology and Allergy, Herlev and Gentofte University Hospital, Hellerup, Denmark; ^3^ Section for Periodontology, Department of Odontology, Faculty of Health and Medical Sciences, University of Copenhagen, Copenhagen, Denmark; ^4^ Department of Clinical Immunology, Aalborg University, Aalborg, Denmark; ^5^ Department of Cardiology, Herlev and Gentofte University Hospital, Hellerup, Denmark; ^6^ Department of Clinical Medicine, University of Copenhagen, Copenhagen, Denmark; ^7^ LEO Foundation Skin Immunology Research Center, Department of Immunology and Microbiology, University of Copenhagen, Copenhagen, Denmark

**Keywords:** psoriasis, autoimmunity, anti-microbial peptides, citrullination, IFN-γ, neutrophil extracellular traps

## Abstract

**Background:**

The DNA-binding peptide LL37 is a suspected autoantigen in psoriasis. It can be found in neutrophil extracellular traps (NETs) which have been suggested to play a role in the pathogenesis of the disease. Citrullination, the conversion of peptidyl-arginine into peptidyl-citrulline, can be implicated in the formation of NETs. We hypothesized that citrullination increases LL37 immunogenicity and that NETs are a source of LL37.

**Objectives:**

We aimed to characterize cytokine responses of B cells and T cells to native and citrullinated LL37 (citLL37) and determine the prevalence and composition of circulating NETs in patients with psoriasis and healthy blood donors (HDs).

**Methods:**

Mononuclear cells (MNCs) and serum were isolated from 20 HDs and 20 patients with psoriasis. The MNCs were stimulated with native LL37 and citLL37 and the proportion of cytokine-positive B cells and T cells was determined by flow cytometry. Circulating antibodies against native LL37 and citLL37 as well as circulating NETs were measured by ELISA, as was the content of LL37, citLL37, and IgG in the NETs.

**Results:**

CitLL37, but not native LL37, induced IFN-γ-production by T cells and B cells from psoriasis patients, as well as IL-10-production by the patients’ CD4^+^ T cells. Serum from 40% of patients and 55% of HDs contained circulating NETs, of which 63% and 27%, respectively, contained LL37. Only two patients had NETs containing citLL37 and IgG antibodies were found in NETs from three patients and one HD. *Post-hoc* analysis of the cytokines produced by B cells and T cells after stimulation with citLL37 revealed two clusters of patients consisting of 10 high-responders and 9 low-responders. The high-responders were those that had circulating NETs in combination with an earlier age of onset of the disease.

**Conclusion:**

Citrullinated but not native LL37 elicits IFN-γ-responses by T cells and B cells from psoriasis patients, particularly those with circulating NETs and early disease onset, suggesting a role of citLL37 as an autoantigen in this subgroup of patients.

## 1 Introduction

With a prevalence of 1.5% in Western Europe, psoriasis is one of the most frequent chronic autoimmune diseases (1). It is characterized by an abnormal proliferation of keratinocytes and migration of various leukocyte populations, including neutrophils, T cells, and B cells, to the skin (2).

Citrullination (deimination) is a posttranslational protein modification, in which peptidyl-arginine is converted into peptidyl-citrulline by peptidyl arginine deiminases (PADs). The majority of patients with rheumatoid arthritis produce anti-citrullinated protein antibodies (3, 4), and we have recently shown that citrullination of myelin basic protein influences its antigenicity in the context of multiple sclerosis (5).

The human cathelicidin LL37 is a suspected autoantigen in psoriasis, that has been shown to elicit T-cell proliferation and IFN-γ production (6). It is abundant in inflamed skin, while healthy skin normally only contains the precursor molecule CAP-18 (7). LL37 is primarily produced by neutrophils, which dominate psoriatic plaque lesions (8), but can also be secreted by keratinocytes, monocytes and macrophages (9); all of which express PADs (10, 11). Antibodies against citrullinated LL37 (citLL37) have been found in some patients with psoriatic arthritis (12), but it is not known whether citrullination enhances immune responses to LL37 in cutaneous psoriasis.

Neutrophils produce neutrophil extracellular traps (NETs) as a mechanism of anti-bacterial defense, but NETs also play a role in the pathogenesis of several autoimmune diseases, including systemic lupus erythematosus (13) and rheumatoid arthritis (14). Increased NET formation has been found in psoriasis (15), and NETs promote inflammatory responses in an imiquimod-induced psoriasis-like mouse model (16). In general, NETs are composed by a structural framework of DNA embroidered with different proteins, including myeloperoxidase (MPO), histones and elastase (17, 18). LL37 readily binds to DNA and has been found associated with NETs in several studies (18–20).

We hypothesized that citrullination of LL37 amplifies immune responses to this putative autoantigen in psoriasis, and we aimed to clarify if NETs from patients with psoriasis contain LL37 in its native and/or citrullinated forms, thereby serving as a scaffold for delivery of this autoantigen to antigen-presenting cells.

## 2 Materials and methods

### 2.1 Study population

This study included 20 healthy donors (HDs) and 20 patients with psoriasis recruited at the Department of Dermatology and Allergy, Copenhagen University Hospital, Herlev and Gentofte with characteristics shown in **Table 1**. None of the patients included were diagnosed with psoriatic arthritis. Collection of blood from both groups was approved by the Ethical Committee for the Capital Region of Denmark (nr. H-19031332) and written informed consent was obtained from all the participants. All patients were naïve for systemic psoriasis treatment, excluding one patient treated with methotrexate and brodalumab with a 6-week washout period prior to recruitment. All patients stopped any topical treatment two weeks prior to recruitment and did not receive phototherapy in the 3 months prior to sample collection.

**Table 1:** Study population demographics and clinical data.

	HDs (n=20)	Psoriasis (n=20)	p-value
**Age, years, mean ± SD**	36.4 (16.4)	44.1 (18.8)	0.18
**Female, n (%)**	9 (45%)	8 (40%)	1
**BMI, kg/m^2^, mean ± SD**	24.9 (4.7)	24.7 (3.2)	0.87
**PASI, mean ± SD**	–	7.3 (5.2)	–
**Age at psoriasis onset, years, mean ± SD**	–	21.5 (14.15)	–
HLA-C*06:02:01 carriers, n (%)	–	8 (40%)	–

HDs, healthy donors; BMI, body mass index; PASI, psoriasis area and severity index; HLA, human leukocyte antigen.

Continuous variables were compared between groups with the Welch Two sample t-test. Categorical variables were compared between groups with a x^2^-test.

### 2.2 Serum isolation

Whole blood was collected in clot activator tubes (cat. 368815; BD Vacutainer®) and spun at 1200 x g for 10 minutes at 4°C. Serum was then aliquoted and stored at -80°C until further use. All aliquots were thawed only once before use.

### 2.3 Isolation of mononuclear cells

Blood was collected in BD CPT™ tubes (cat. 362780; BD Vacutainer®) which contain both sodium heparin as anticoagulant and a liquid density medium that allows in-tube mononuclear blood cell separation. Subsequently, the tubes were spun at 1500 x g for 20 minutes at room temperature. After centrifugation, mononuclear cells (MNCs) were collected, resuspended in PBS + 2% fetal bovine serum (FBS) (v/v) and washed twice at 300 x g for 10 minutes. Cells were counted with a NucleoCounter® NC-100™ device (cat. 900-0004; Chemometec) and resuspended in RPMI 1640 medium (cat. #01-106-1a; Lonza) supplemented with 30% FBS (v/v) and 10% DMSO (v/v) for cryopreservation. Aliquots of 5x10^6^ cells were frozen overnight in CoolCell® LX boxes (cat. BCS-405PK; Biocision) at -80°C and transferred to liquid nitrogen tanks the next day.

Isolation of MNCs from one HD and one patient with psoriasis yielded less than 5x10^6^ cells; both were excluded from the experiments involving cell cultures.

### 2.4 Antigens

Lyophilized native LL37 (LLGDFFRKSKEKIGKEFKRIVQRIKDFLRNLVPRTES) and citLL37 (LLGDFF[Cit]KSKEKIGKEFK[Cit]IVQ[Cit]IKDFL[Cit]NLVP[Cit]TES) (cat. SP-LL37-1 and SP-5380-1, Innovagen) were resuspended in sterile water at a concentration of 1 mg/ml. Aliquots were stored at -80°C. Lyophilized tetanus toxin (cat. 582243, Calbiochem) was resuspended in sterile water at a concentration of 50 μg/ml and stored at 4°C.

### 2.5 Cell cultures

MNC aliquots were thawed and resuspended in RPMI 1640 supplemented with 10% (v/v) normal human serum (NHS) (cat. H4522, Sigma-Aldrich). Cells were then washed in PBS + 2% FBS (v/v) and counted using a NucleoCounter® NC-100™ device. 250,000 MNCs were added to each well in a final volume of 200 μl RPMI 1640 supplemented with 0.1% (v/v) gentamycin and 10% (v/v) NHS in 96-well, round-bottomed plates. Cells were cultured in the presence or absence of 5 μg/ml of native LL37, citLL37, or 0.5 μg/ml tetanus toxin for 29 hours. At that timepoint, cells were re-stimulated with the same concentrations of either native LL37, citLL37, or tetanus toxin used for initial stimulation. At 32 hours, a mix of brefeldin A + monensin (cat. 555029 and 554724, respectively, BD Biosciences) was added, diluted 1:1000 and 1:1500, respectively, to allow intracellular accumulation of cytokines for 16 hours. All samples were measured as duplicates during the optimization process to assess timepoints for cytokine measurement and eligible concentrations of LL37 and citLL37. Once the experimental conditions were established, the samples were run in singlets due to limitations in the amounts of cells available.

### 2.6 Analysis of cytokine production

After 48 hours of culturing, plates were spun for 5 minutes at 300 x g, 4°C. MNCs were washed with 200 μl PBS + 2% FBS (FACS-PBS) and spun again as described above. Cells were resuspended in a mix of 1 μl immunoglobulin for intravenous use (IvIg, cat. 034401, CSL Behring) and 2 μl mouse serum (MS, cat. M5905, Sigma Aldrich) and subsequently, 20 μl of a mix of pre-titrated antibodies against extracellular markers was added as described in **Table 2** for 30 minutes at 4°C. MNCs were then washed and resuspended in 100 μl of fixation/permeabilization solution (cat. 51.2090KZ, BD Biosciences). After 20 minutes of incubation at 4°C, cells were washed twice in 200 μl BD Perm/WashTM Buffer (cat. 51-2090KZ, BD Biosciences) and resuspended in 3 μl of MS + IvIg and 20 μl of a mix of pre-titrated antibodies against the cytokines captured inside the cells as shown in **Table 2**. MNCs were re-incubated for 30 minutes at 4°C and washed in BD Perm/WashTM Buffer. Subsequently, they were resuspended in 200 μl PBS, and 80,000 cells were acquired using an AttuneTM NxT flow cytometer (Thermo Fisher Scientific). The gating strategy followed for the identification of cytokine-producing MNC populations is shown in **Supplementary Figure 1**. The percentages of CD4^+^ T, CD8^+^ T, and B cells as well as the absolute number of cells in the gates in relation to the total amount of events acquired are presented in **Supplementary Table 1**. Also shown in that table are the percentages of cytokine-positive cells in relation to their parental population as well as the absolute number of cells in the gates.

**Table 2:** Antibodies used for identification of MNC populations producing cytokines.

Antibodies against surface markers and staining of live cells	Volume added per well (cat. nr., manufacturer)	Antibodies against cytokines	Volume added per well (cat. nr., manufacturer)
**Anti-CD45 PerCP**	1 μl (cat. 587513, BD Biosciences)	**Anti-IL-6 FITC**	10 μl (cat. 340526, BD Biosciences)
**Anti-CD8 PE-Cy7**	0.5 μl (cat. 557746, BD Biosciences)	**Anti-TNF-α PE-eFluor 610**	0.5 μl (cat. 61-7349-42, Thermo Fisher Scientific)
**Anti-CD4 Alexa Fluor 700**	0.5 μl (cat. 566318, BD Biosciences)	**Anti-IL-17 PE**	0.5 μl (cat. 560436, BD Biosciences)
**Anti-CD3 Brilliant Violet 510**	1 μl (cat. 564713, BD Biosciences)	**Anti-IL-22 APC**	1 μl (cat. 366706, BioLegend)
**Anti-CD19 Brilliant Violet 421**	0.5 μl (cat. 562440, BD Biosciences)	**Anti-IL-10 Brilliant Violet 711**	0.5 μl (cat. 564050, BD Biosciences)
**LIVE/DEAD Fixable Near-IR**	4 μl, 1:40 dilution (cat. L34976, Thermo Fisher Scientific)	**Anti-IFN-γ Brilliant Violet 605**	1 μl (cat. 562974, BD Biosciences)

### 2.7 Measurement of cytokines in culture supernatants

The concentrations of TNF-α (analyzed by means of bead and antibody set with cat. 171BA015M, Bio-Rad), IL-10 (171BA004M, Bio-Rad), IL-17 (171BA005M, Bio-Rad), IFN-γ (171BA013M, Bio-Rad), and IL-22 (171BA008M, Bio-Rad) in undiluted culture supernatants were analyzed by Luminex assays (reagent kit and standards; cat. 171304090M and 171DA0001, Bio-Rad) using a Bio-Plex 200 reader (Bio-Rad) according to the manufacturer’s protocol. The upper and lower limits of quantification (ULOQ and LLOQ, respectively) for each assay were: ULOQ/LLOQ (TNF-α): 4.68 and 0.3 pg/ml; ULOQ/LLOQ (IL-10): 12.92 and 3.2 pg/ml; ULOQ/LLOQ (IL-17): 25.92 and 3.1 pg/ml; ULOQ/LLOQ (IFN-γ): 11.38 and 0.7 pg/ml; and ULOQ/LLOQ (IL-22): 41.57 and 2.5 pg/ml.

### 2.8 Measurement of circulating antibodies against LL37 and citLL37

Nunc amino immobilizer plates C8 (cat. 436023; Thermo Fisher) were coated for 1 hour shaking with 100 μl of a 2 μg/ml dilution of native LL37 or citLL37 in 100 mM sodium carbonate buffer, pH 9.6. Subsequently, wells were washed three times with PBST 0.05%, and 100 μl of 10 mM ethanolamine (cat. E9508, Sigma-Aldrich) diluted in coating buffer was added for 1 hour. After three further washes with PBST 0.05%, 100 μl of serum samples, diluted 1:25 in PBST 0.05%, was added. Plates were incubated for 1 hour, shaking. After three washes, 100 μl of an anti-human IgG antibody conjugated to horseradish peroxidase (HRP) (cat. P0214, Dako), diluted 1:15000 in PBST 0.05%, was added for one hour, shaking. Wells were washed three times more, and 100 μl of 1-Step Ultra TMB ELISA Substrate solution (cat. 34029, Thermo Fisher) was added. The colorimetric reaction was stopped with 0.5 M H_2_SO_4_, and optical density (OD) was measured at 450 and 540 nm using a SPECTROstar nano Microplate Reader (BMG LabTech).

All samples were run in duplicates and uncoated wells were used as negative controls. Net OD values were obtained after subtraction of the signal from blank wells.

### 2.9 ELISAs for circulating NETs and their composition

Flat-bottomed, 96-well SH plates (cat. 269620, Thermo Fisher) were coated overnight at 4°C with 100 μl of an anti-MPO antibody (cat. 0400-0002, BioRad) at a final concentration of 2 μg/ml diluted in carbonate-bicarbonate buffer (15.7 mM and 34.3 mM, respectively), pH 9.6. The next day, wells were washed 3 times with 200 μl PBS + 0.2% (v/v) Tween-20 (PBST 0.2%) and blocked with 100 μl PBST 0.2% + 5% (v/v) milk (cat. M7409, Merck) for 2 hours. Wells were then washed 3 times with PBST 0.2% and 100 μl of serum was added to each well diluted 1:25 in PBST 0.2% + 1% probumin. Plates were incubated at 4°C overnight and the next day wells were washed 5 times as described above. Subsequently, 100 μl of an antibody against DNA conjugated to HRP (cat. 11544675001, Roche) was added at a 1:100 dilution in PBST 0.2% + 1% probumin.

For analysis of native LL37, citLL37 and IgG associated with NETs, ELISA plates were coated, blocked, and incubated with serum as described above but the detection antibody systems were those described in **Table 3**, all in a total volume of 100 μl. Detection of human serum albumin in NETs was used as a negative control.

**Table 3:** Antibodies used for detection of LL37, citLL37 and IgG in circulating NETs.

Primary antibody	Concentration (cat. nr., manufacturer)	Secondary antibody	Concentration (cat. nr., manufacturer)
**Sheep anti-human LL37**	1 μg/ml (cat: AF7497; R&D systems)	**HRP-donkey anti-sheep IgG**	0.067 μg/ml (cat: A16041, ThermoFisher Scientific)
**Mouse anti-citLL37**	0.5 μg/ml (cat: 26739; Cayman Chemicals)	**HRP-goat anti-mouse IgG1**	0.087 μg/ml (cat: A10551, Invitrogen)
**HRP-rabbit anti-human IgG**	0.087 μg/ml (cat: P0214; Dako)		
**Rabbit anti-human serum albumin**	1.33 μg/ml (cat: 109-4133; Rockland)	**HRP-goat anti-rabbit IgG**	0.067 μg/ml (cat: P0448, Dako)

HRP, horseradish peroxidase.

In all cases, after 2 hours of incubation with the HRP-conjugated antibody, wells were washed 5 times with PBST 0.2% and 100 μl of 1-Step Ultra TMB ELISA Substrate solution (cat. 34029, Thermo Fisher) was added. The colorimetric reaction was stopped with 0.5 M H_2_SO_4_, and OD was measured at 450 and 540 nm using a SPECTROstar nano Microplate Reader (BMG LabTech).

All samples were run in duplicates and uncoated wells lacking the anti-MPO antibody, were used as negative controls. Final OD values were obtained after subtraction of the signal from blank wells.

### 2.10 Statistics

Cut-offs for the ELISA assays were calculated using a segmented regression model with breakpoint estimation using the R Studio software (1.4.1717, R Studio, 4.1.2); estimated breakpoints and confidence intervals for each breakpoint estimate are shown in **Supplementary Figures 2** and **3**. Differences between patients and HDs in the frequencies of individuals positive for NETs and positive for NETs containing native LL37, citLL37 and IgG, as well as differences in the NET content between the two patient clusters, were calculated with Fisher’s exact test. Differences between unstimulated samples and native LL37- or citLL37-stimulated samples were evaluated using the Wilcoxon matched-pairs signed-rank test. Differences between the age of disease onset, body mass index, and psoriasis area and severity index of the two patient clusters were assessed using the Mann-Whitney U test. p-values <0.05 were considered statistically significant. The clustering of patients and controls was performed using the ComplexHeatmap package in R Studio applying Ward’s method for hierarchical clustering to the rows (representing the percentage of positive cells for each cytokine produced after citLL37 stimulation). The argument “k_row” was not specified to allow the clustering method to determine the optimal number of clusters in both groups. Graphical representation of the data and statistics were performed with R studio and/or GraphPad Prism 9 software (GraphPad Software, San Diego, CA, USA).

## 3 Results

### 3.1 T-cell responses to native LL37 and citrullinated LL37

**Figure 1:**
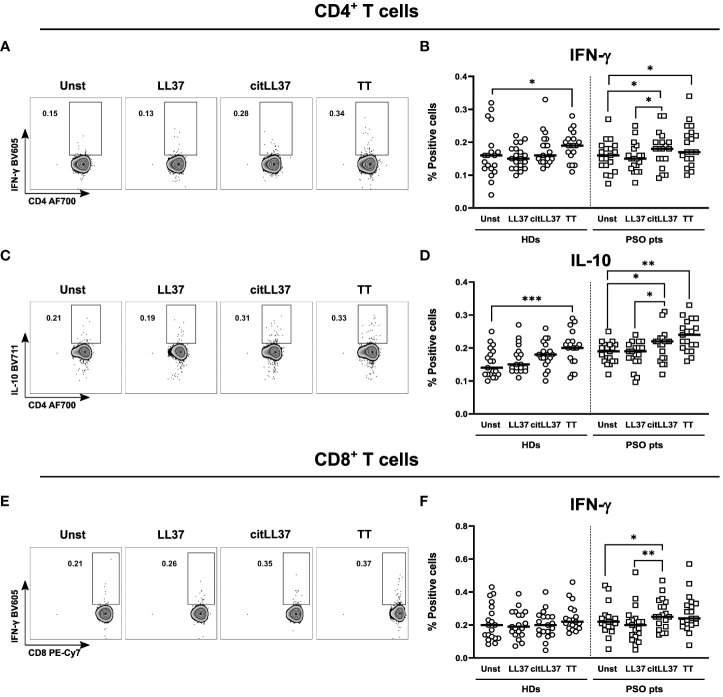
T-cell cytokine production induced by native and citrullinated LL37. MNCs from 19 healthy donors (HDs) and 19 patients with psoriasis (PSO pts) were cultured in the presence or absence of 5 μg/ml of native LL-37 (LL37) or citrullinated LL37 (citLL37). Tetanus toxin (TT) was used as a positive control. After 29 hours, the cells were re-stimulated, and 19 hours later, cytokine production by T cells was analyzed by flow cytometry. **(A)** Dot plots showing IFN-γ producing CD4^+^ T cells for a representative patient, and **(B)** scatter plot showing the percentage of IFN-γ-producing CD4^+^ T cells in cultures from HDs and PSO pts. **(C, D)** Corresponding data for IL-10-producing CD4^+^ T cells and **(E, F)** IFN-γ-producing CD8^+^ T cells. Horizontal bars represent median values. Wilcoxon matched-pairs signed rank test, *p<0.05, **p<0.01, ***p<0.001.

We examined the cytokine responses of CD4^+^ and CD8^+^ T cells to native LL37 and its citrullinated counterpart, citLL37 (**Figure 1**). Tetanus toxin (TT) was used as positive control.

Neither native nor citLL37 induced IFN-γ-producing CD4^+^ T cells in MNC cultures from HDs, while citLL37 induced a significant increase in the proportion of IFN-γ-producing CD4^+^ and CD8^+^ T cells in cultures from patients with psoriasis compared to unstimulated- and native LL37-stimulated cultures (**Figures 1A, B, E, F**). CitLL37 also induced a significant increase in the proportion of IL-10-producing CD4^+^ T cells from patients with psoriasis compared to unstimulated cultures and cultures stimulated with native LL37, and a similar tendency was observed in the HD group (**Figures 1C, D**). We detected very low frequencies of IL-17^+^ CD4^+^ T cells after stimulation of patient and control MNCs with LL-37 or citLL37 (**Supplementary Figure 4**), but citLL37 did induced a significant increase in the proportion IL-17 producing cells from patients with psoriasis compared to unstimulated and LL-37 stimulated cultures. We did not observe induction of IL-22- or TNF-α-producing CD4^+^ T cells in either group.

### 3.2 B-cell responses to native LL37 and citrullinated LL37

**Figure 2:**
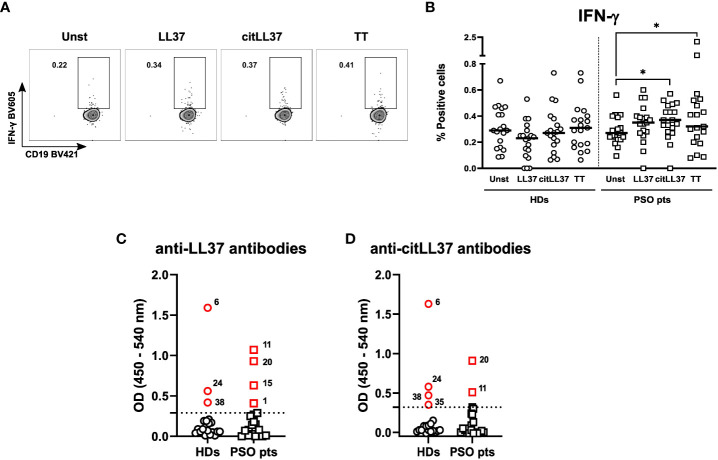
LL37-induced cytokine responses by B cells and circulating levels of anti-LL37 antibodies. MNCs from 19 healthy donors (HDs) and 19 patients with psoriasis (PSO pts) were cultured in the presence or absence of 5 μg/ml of native LL-37 (LL37) or citrullinated LL37 (citLL37). Tetanus toxin (TT) was used as a positive control. After 29 hours, the cells were restimulated, and 19 hours later, cytokine production by B cells was analyzed by flow cytometry. **(A)** Dot plots showing IFN-γ producing B cells from a representative patient, and **(B)** scatter plot showing the percentage of IFN-γ-producing B cells in cultures from HDs and PSO pts. Horizontal bars represent median values. Wilcoxon matched-pairs signed rank test, *p<0.05. **(C, D)** Sera from 20 HDs and 20 PSO pts were assessed for content of antibodies against native LL37 and citLL37 by ELISA. Dotted lines represent the cut-off that defines positive values, as calculated by means of a regression model with segmented relationships. Red symbols represent values above the cut-off. The numbers next to each symbol describe the individual positive for the respective antibodies.

The proportion of IFN-γ-producing B cells in MNC cultures from patients increased upon stimulation with both forms of LL37, albeit only significantly after stimulation with citLL37 (**Figures 2A, B**). TT was used as a positive control. B cells producing IL-6, TNF-α, or IL-10 were not detected in either of the study groups.

Three HDs (15%) and four patients (20%) showed antibody reactivity against native LL37 (**Figure 2C**). Of these, all three HDs and two of the patients also tested positive for antibodies against citLL37, while one HD displayed antibody reactivity against the citrullinated peptide only (**Figure 2D**).

### 3.3 LL37- and citrullinated LL37-induced cytokine secretion by MNCs

**Figure 3:**
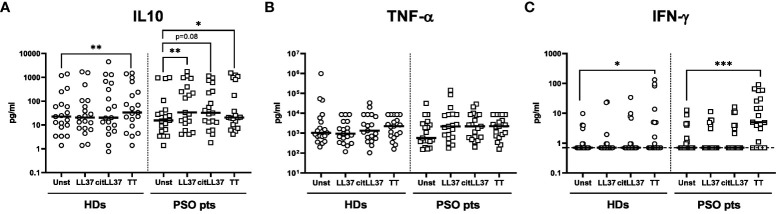
MNC cytokine secretion induced by native LL37 and citLL37. MNCs from 19 healthy donors (HDs) and 19 patients with psoriasis (PSO pts) were cultured in the presence or absence of 5 μg/ml of native LL37 (LL37) or citrullinated LL37 (citLL37) for 48 hours, and secretion of **(A)** IL-10, **(B)** TNF-α and **(C)** IFN-γ into the culture supernatants was examined by a Luminex assay. Tetanus toxin (TT) was used as a positive control. Measurements below the lower level of detection of the IFN-γ assay (0.7 pg/ml) were assigned this value. Horizontal bars represent medians. *p<0.05, **p<0.01, ***p<0.001 (Wilcoxon matched-pairs signed rank test).

Native LL37 induced secretion of IL-10 by MNCs isolated from patients with psoriasis, and a similar trend was observed after stimulation with citLL37 (**Figure 3A**). Both forms of LL37 tended to induce secretion of TNF-α by MNCs from patients with psoriasis, but not by MNCs from HDs (**Figure 3B**). IFN-γ was only detected in some culture supernatants and did not show any distinct pattern (**Figure 3C**). TT was used as a positive control. The levels of IL-17 and IL-22 in the culture supernatants were constantly below the detection limit of the assays.

### 3.4 Composition of circulating NETs

Having established that LL37 is capable of inducing cytokine production by T cells and B cells, we examined if circulating NETs contain LL37.

**Figure 4:**
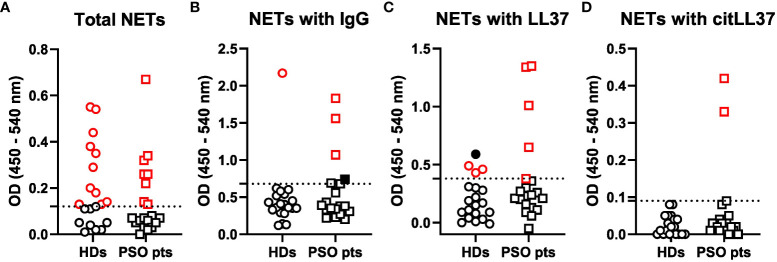
Circulating NET levels and composition. Sera from 20 healthy donors (HDs) and 20 patients with psoriasis (PSO pts) were added to ELISA plates coated with an anti-MPO antibody to capture NETs. Subsequently, antibodies against **(A)** DNA, **(B)** IgG, **(C)** native LL37 (LL37) and **(D)** citrullinated LL37 (citLL37) were used as detection antibodies in combination with HRP-conjugated secondary antibodies, and optical density (OD) was measured at 450 and 540 nm. Dotted lines represent the cut-off between positive and negative values, as calculated by means of a regression model with segmented relationships. Red symbols represent values above the cut-off, and filled, black symbols represent subjects who tested negative in **(A)** and therefore were regarded as false positives.

A cut-off OD value to differentiate NET-positive from NET-negative samples was calculated (**Supplementary Figure 2**), and on this basis, 8 out of 20 patients with psoriasis (40%) and 11 out of 20 healthy donors (HDs) (55%) tested positive for circulating NETs (**Figure 4A**).

The NETs from three out of the eight NET-positive patients (38%) versus one out of the eleven NET-positive HDs (9%) showed attachment of IgG (**Figure 4B**), and the NETs from five patients with psoriasis (63%) versus three HDs (27%) contained native LL37 (**Figure 4C**). CitLL37 was only found in NETs from two individuals, who were both patients with psoriasis (25%) (**Figure 4D**).

### 3.5 Clustering of patients and controls according to citLL37 responses

**Figure 5:**
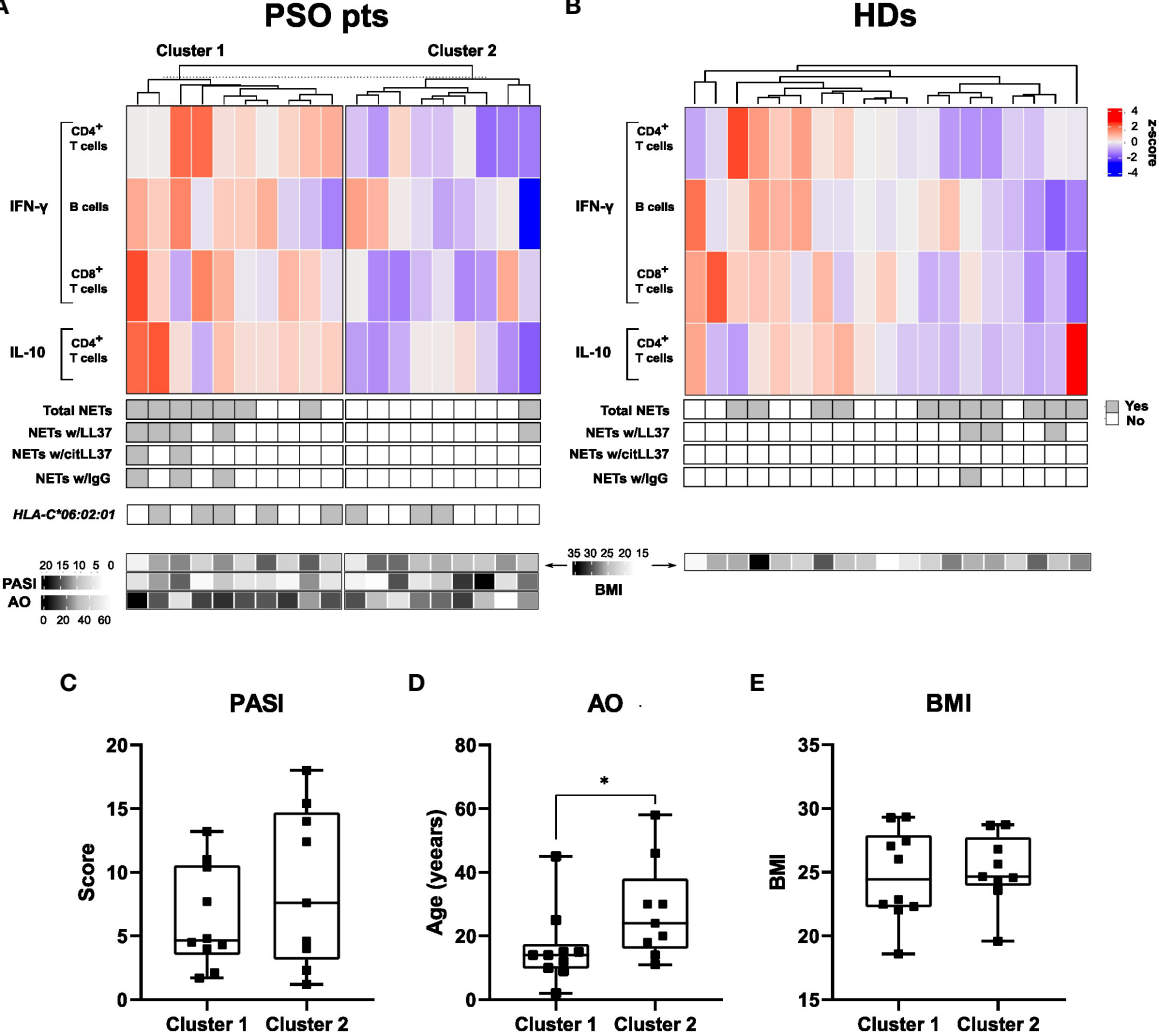
Clusters of subjects defined on the basis of citLL-37-induced cytokine production. **(A)** Top: Heatmap showing the proportion of CD4^+^ T cells, B cells and CD8^+^ T cells producing IFN-γ or IL-10 after stimulation of MNCs from psoriasis patients (PSO pts) with citLL-37 (z-score). Middle: Panels showing the presence of circulating NETs and their content of native LL37 (LL37), citrullinated LL37 (citLL37) and IgG together with the expression of the psoriasis risk allele HLA-C*06:02:01. Bottom: Heatmap showing body mass index (BMI, top row), psoriasis area and severity index (PASI) (middle row) and age of disease onset (AO) for the individual patients. **(B)** The corresponding data for healthy donors (HDs). **(C)** Differences between clusters with respect to PASI, **(D)** AO, and **(E)** BMI. Mann-Whitney U test, *p<0.05.

Based on the T- and B-cell IFN-γ and IL-10 responses to citLL37 in patients with psoriasis, we identified two distinct clusters of patients: ten high-responders (cluster 1) and nine low-responders (cluster 2) (**Figure 5A**). No clear clustering was observed among HDs (**Figure 5B**). The majority of patients (70%) in cluster 1 had circulating NETs containing either native LL37, citLL37 and/or IgG, versus only one patient (11%) in cluster 2 (*p=0.019*).

Neither the cytokine responses to citLL37 nor the presence of circulating NETs correlated with the carriage of the risk allele HLA-C*06:02:01 in the patient group. HLA-C*06:02 is the strongest susceptibility allele regarding genetic risk factors (21), with roughly 50% of patients with psoriasis carrying it (22).

Clinically, patients in cluster 1 had lower age at psoriasis onset than patients in cluster 2 (**Figure 5D**). The BMI and PASI varied highly within both clusters and did not differ significantly between them (**Figures 5C, E**).

## 4 Discussion

Citrullination is known to render self-proteins immunogenic in rheumatoid arthritis, and we have recently shown that citrullination of myelin basic protein influences its antigenicity in the context of multiple sclerosis (5). In the present study, we examined the cytokine responses to native and citrullinated LL37 of T cells and B cells from patients with psoriasis and HDs, and if citLL37 or LL37 associates with circulating NETs in either of the two groups.

We found that citLL37, unlike native LL37, induced IFN-γ production by both T-helper and cytotoxic T cells in MNC cultures from patients with psoriasis, but not in cultures from HDs. In contrast, *Lande et al.* found ample IFN-γ production by T cells after stimulation with native LL37, but they did not examine the response to citrullinated LL37 (6). The discrepancies between our findings and those of *Lande et al.* may rely on differences in the experimental set ups. In addition to Th1 cells and cytotoxic T cells, Th17 cells are known to play essential roles in the pathogenesis of psoriasis, but the frequencies of CD4^+^ IL-17^+^ cells observed in our study were too low to draw reliable conclusions. In psoriasis, IFN-γ contributes to the IL-17/IL-23 pathogenic axis by promoting the production of IL-23 by tissue-resident dendritic cells and macrophages (23) and is thereby an inducer of Th17-cell differentiation (24).

CitLL37 induced IL-10-producing CD4^+^ T cells in patients with psoriasis, exclusively, and IL-10 was detected in the culture supernatants after stimulation of MNCs from patients with psoriasis with LL37 as well as with citLL37. This may be a compensatory response in the case of citLL37 stimulation, since IL-10 counteracts the production of pro-inflammatory cytokines and prevents “overshoot” of the pro-inflammatory response induced by the modified peptide.

We observed that B cells from patients with psoriasis, but not those from HDs, also respond to challenge with citLL37 with production of IFN-γ. Patients with psoriasis did not differ from HDs with respect to production of antibodies to native or citrullinated LL37, which is in accordance with previous reports (12, 25).

Since LL37 binds to DNA, and *Hu et al.* have reported increased proportions of NETotic cells in peripheral blood from patients with psoriasis (15), we speculated that NETs may be a source of this putative autoantigen and facilitate its uptake by antigen-presenting cells. We found, however, that the proportion of patients with psoriasis and HDs with circulating NETs was similar. The discrepancy between our results and those of *Hu et al.* may be attributed to more disease severity in their patient group compared to ours, and/or on technical aspects regarding measurement of NETs. While they used a DNA stain to quantify the number of traps by fluorescence microscopy, we assessed NET levels with a commonly used ELISA (26, 27), which is based on the recognition of both MPO and DNA. We did observe that both native LL37 and citLL37, as well as IgG antibodies, can be found associated with circulating NETs in psoriasis, but our study was underpowered to show significant differences from the corresponding frequencies in HDs.

Stratification of the patients on the basis of T-cell and B-cell cytokine responses to citLL37 revealed two distinct subgroups. Interestingly, 70% of the patients in the cluster of high-responders had circulating NETs containing LL37, citLL37 and/or IgG versus only one patient among the low-responders. It remains to be investigated if the production of IFN-γ by B cells and T cells characteristic of the high-responders directly stimulates NETosis, but previous observations of IFN-γ production by macrophages (28) and NK cells (29) inducing NET formation suggest that this may be the case. The median age of onset of psoriasis was significantly lower for the high-responders than for the low-responders. Notably, *Henseler et al.* previously described two types of psoriasis based on an early (<40 years) or late (≥40 years) onset of the disease (30) and more recent data by *Bergboer et al.* showed a genotypic distinction between pediatric-onset (<18 years) and adult onset (≥18 years) psoriasis (31). These findings are within the range of ours of the median ages of onset of citLL37 high-responders and low-responders. However, both studies found associations between early onset of the disease and the HLA-C*06:02:01 risk allele and/or other risk loci of the disease, such as *ERAP1* and *IL23R*, whereas our group of high-responders was not enriched in carriers of HLA-C*06:02:01.

Segregation of psoriasis patients into two or more subgroups based on immunological reactions may have clinical implications, in that these subgroups may respond differently to various treatments. Particularly, since the two subgroups of patients responded differently to citLL37, inhibitors of citrullination may have a beneficial effect in high-responders to citLL37.

A limitation of this explorative study was the small sample size. Additionally, cellular immune responses were analyzed in peripheral MNCs, which may not be representative of skin tissue resident and psoriatic plaque-infiltrating immune cells.

In conclusion, we show that T-helper cells, cytotoxic T cells and B cells from a subgroup of patients with psoriasis respond to challenge with citLL37, but not native LL37, with production of IFN-γ accompanied by production of IL-10 by CD4^+^ T cells. This subgroup has relatively early psoriasis onset, and it comprises individuals with circulating NETs containing native LL37 and, in some cases, citLL37.

## Data availability statement

The raw data supporting the conclusions of this study will be made available by the corresponding author upon reasonable request.

## Ethics statement

The studies involving humans were approved by Ethical Committee for the Capital Region of Denmark. The studies were conducted in accordance with the local legislation and institutional requirements. The participants provided their written informed consent to participate in this study.

## Author contributions

Conceptualization: MM and CN. Data curation: AK-H and NL. Formal analysis: MM. Funding acquisition: MM, PH, NØ, and CN. Investigation: MM and RS. Methodology: MM and LM. Project administration: LS and CN. Resources: AK-H, NL, PH, and CN. Supervision: LS and CN. Validation: MM. Visualization: MM. Writing – Original draft preparation: MM and CN. Writing – Review and editing: AK-H, LM, RS, NL, PH, NØ and LS. All authors contributed to the article and approved the submitted version.
