# Supplementary material for: Characterization of circulating extracellular traps and immune responses to citrullinated LL37 in psoriasis
Source: Front Immunol. 2023 Dec 19;14:1247592. doi: 10.3389/fimmu.2023.1247592 (PMC10762777; doi:10.3389/fimmu.2023.1247592)
Supplement: Supplementary file 1 [file DataSheet_1.docx]

Supplementary Material

Characterization of circulating extracellular traps and immune responses to citrullinated LL37 in psoriasis

**María Teresa Martín Monreal^1^, Amanda Kvist-Hansen^2^, Laura Massarenti^1,3^, Rudi Steffensen^4^, Nikolai Loft^2^, Peter Riis Hansen^5,6^, Niels Ødum^7^, Lone Skov^2,6^ and Claus H. Nielsen^1,3^**

*** Correspondence:**Claus H. Nielsen
claus.henrik.nielsen@regionh.dk

# Supplementary Data

##
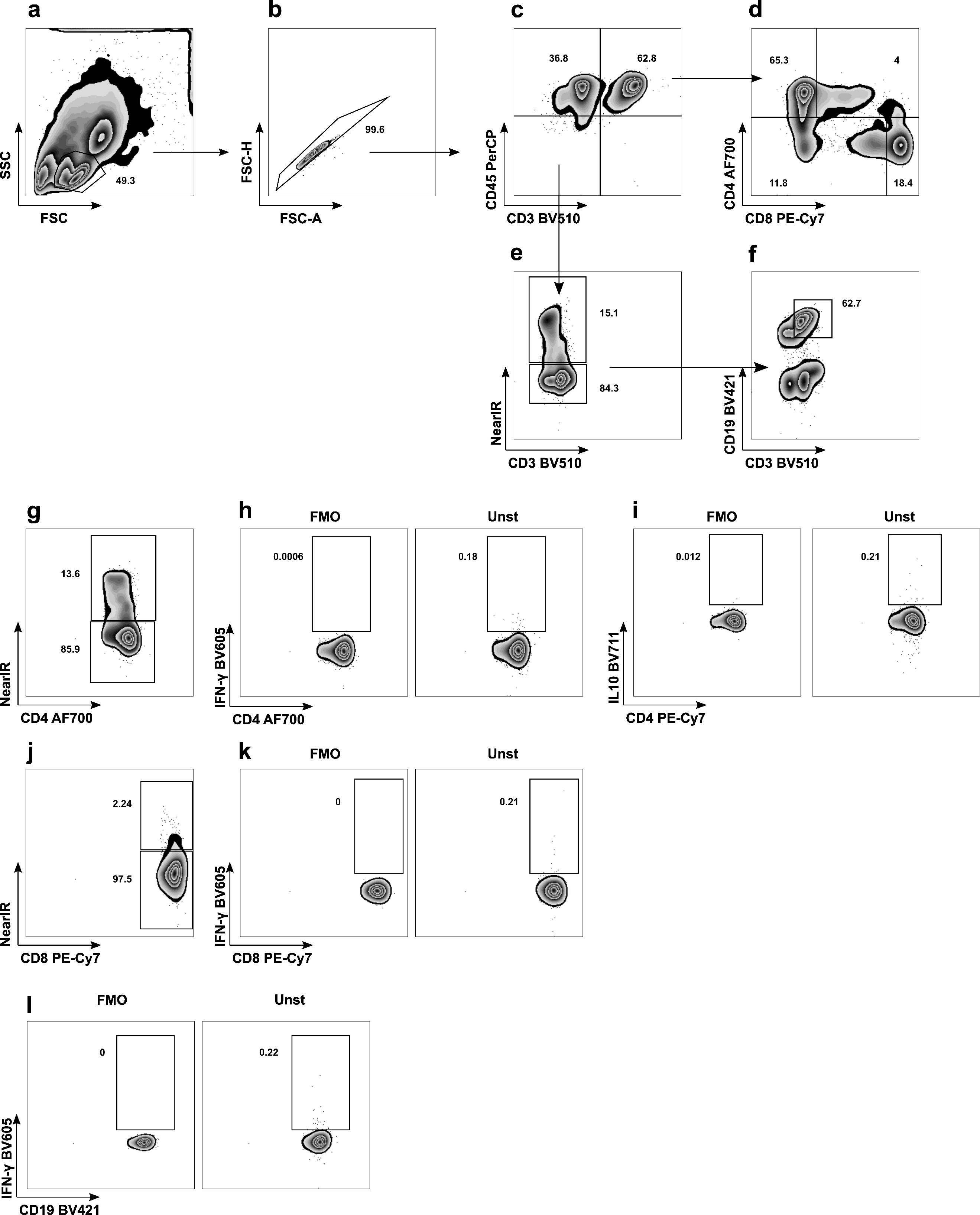
Supplementary Figure 1

**Supplementary figure 1. *Gating strategy for the detection of cytokine-producing B and T cells*.** **(a)** Morphological gating on the lymphocyte population, **(b)** exclusion of doublets by means of forward side scatter (FSC)-A and FSC-H, and **(c)** identification of CD45^+^ CD3^+^ (T cells) and CD45^+^ CD3^-^ populations. **(d)** Gating of CD4^+^ and CD8^+^ T cells from the CD45^+^ CD3^+^ population. **(e, f)** Gating of live CD19+ B cells (high). **(g)** Separation into live and dead CD4^+^ T cells. **(h, i)** Gating of CD4^+^ T cells producing IFN-γ and IL-10, respectively, based on a fluoresce minus one (FMO) sample. **(j)** Separation into live and dead CD8^+^ T cells. **(k, l)** Identification of IFN-γ producing CD8^+^ T cells and B cells, respectively, based on a FMO control. A logarithmic scale was used for both the x and y axis of all graphs.

## Supplementary Figure 2


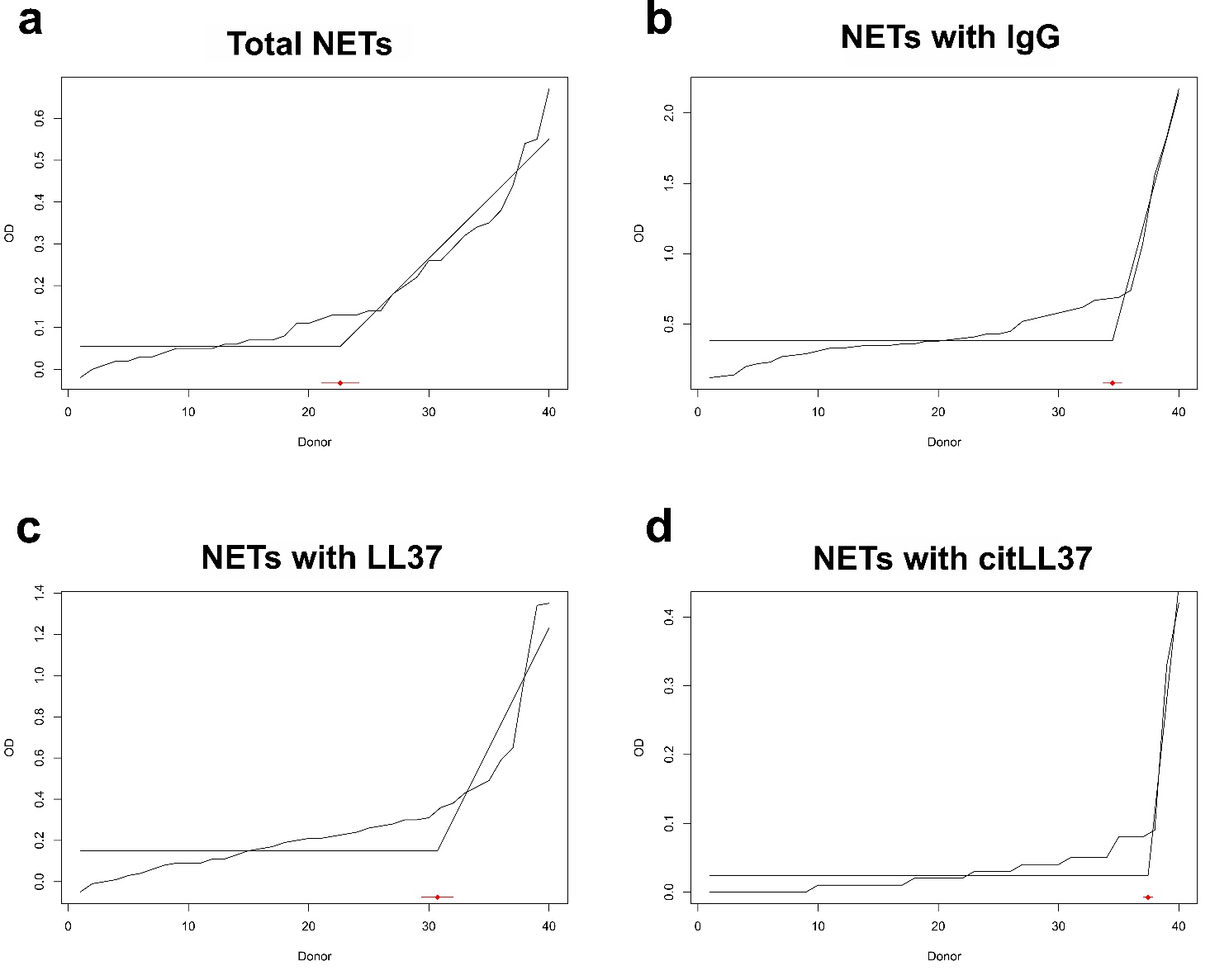


**Supplementary Figure 2. *NET ELISA cut-off calculation with a linear model with segmented relationships*.** To define positive OD values for detection of circulating NETs and study of their composition, a linear model with segmented relationships was used. OD values were listed in ascending order, and positions for break points were calculated. Red dots and lines represent the break point estimate and the confidence interval (CI) for such estimate. Graphical representation of the segmented model for the assay detecting: **(a)** total amount of circulating NETs with a break point estimate in position 23 which is equivalent to an OD value of 0.12, CI (21.08, 24.19), **(b)** the presence of IgG in circulating NETs with a break point estimate in position 34 which is equivalent to an OD value of 0.68, CI (33.71, 35.26), **(c)** the presence of LL37 in circulating NETs with a break point estimate in position 31 which is equivalent to an OD value of 0.36, CI (29.38, 32.04), and **(d)** the presence of citLL37 in circulating NETs with a break point estimate in position 38 which is equivalent to an OD value of 0.09, CI (37.06, 37.84).

## Supplementary Figure 3


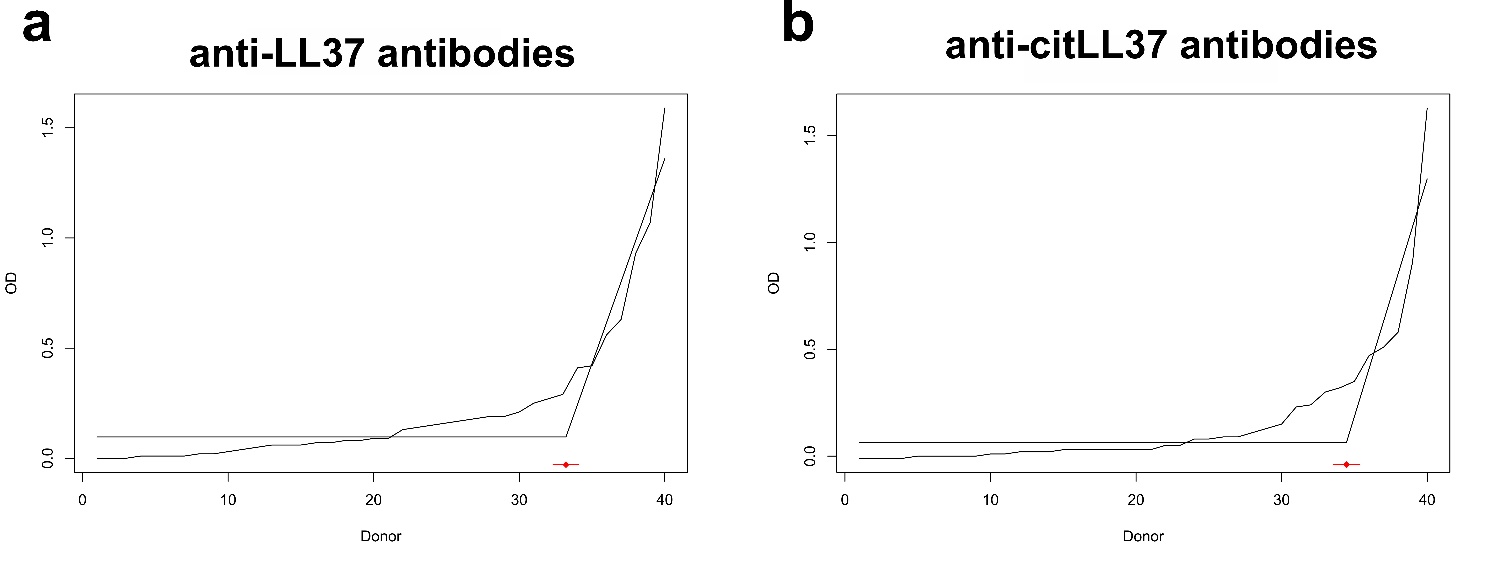


**Supplementary Figure 3. *Antibody ELISA cut-off calculation with a linear model with segmented relationships*.** To identify positive OD values for detection of circulating antibodies against LL37 and citLL37, a linear model with segmented relationships was used. OD values were listed in ascending order and positions for break points were calculated. Red dots and lines represent the break point estimate and the confidence interval (CI) for such estimate. Graphical representation of the segmented model for: **(a)** anti-LL37 antibodies with a break point estimate in position 33 which is equivalent to an OD value of 0.29, CI (32.33, 34.1), and **(b)** anti-citLL37 antibodies with a break point estimate in position 34, which is equivalent to an OD value of 0.32, CI (33.54, 35.37).

## Supplementary Figure 4


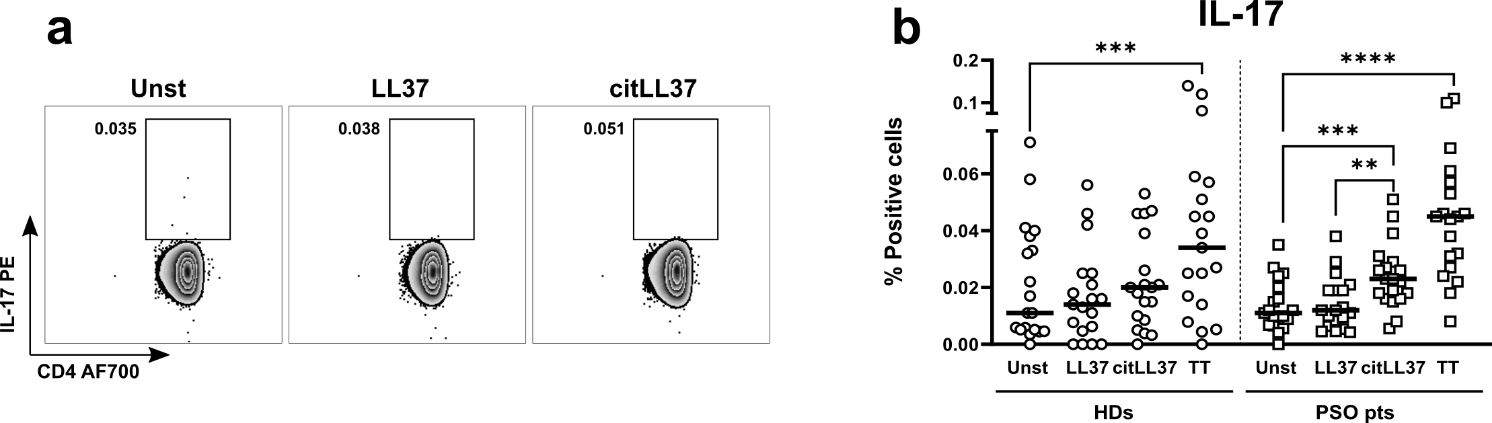


**Supplementary Figure 4. *IL-17 cytokine production of CD4^+^ T cells induced by native and citrullinated LL37*.** MNCs from 19 healthy donors (HDs) and 19 patients with psoriasis (PSO pts) were cultured in the presence or absence of 5 μg/ml of native LL-37 (LL37) or citrullinated LL37 (citLL37). Tetanus toxin (TT) was added as a positive control. After 29 hours, the cells were re-stimulated, and 19 hours later, cytokine production by T cells was analysed by flow cytometry. **(a)** Dot plots showing IL-17 producing CD4^+^ T cells for a representative patient, and **(b)** scatter plot showing the percentage of IL-17-producing CD4^+^ T cells in cultures from HDs and PSO pts. Horizontal bars represent median values. Wilcoxon matched-pairs signed rank test, **p<0.01, ***p<0.001, ****p<0.0001.

## Supplementary Table 1. Descriptive statistics for flow cytometry analysis.

|  | % of total (median, range) (n=40) | No. cells (median, range) (n=40) |
| --- | --- | --- |
| Live CD4 T cells | 23 (10, 46) | 20816 (10401, 36906) |
|  | |  |
|  | % of live CD4^+^ T cells (median, range) (n=20) | No. cells (median, range) (n=20) |
| IFN-γ Unst | 0.16 (0.074, 0.2) | 32 (15, 66) |
| IFN-γ LL37 | 0.15 (0.077, 0.25) | 32 (13, 59) |
| IFN-γ citLL37 | 0.18 (0.091, 0.28) | 42 (14, 75) |
| IFN-γ TT | 0.17 (0.1, 0.34) | 43 (11, 75) |
|  | |  |
| IL-10 Unst | 0.19 (0.12, 0.25) | 40 (20, 86) |
| IL-10 LL37 | 0.19 (0.096, 0.24) | 39 (16, 88) |
| IL-10 citLL37 | 0.22 (0.12, 0.31) | 47 (18, 107) |
| IL-10 TT | 0.24 (0.16, 0.33) | 51 (31, 94) |

|  | % of total (median, range) (n=40) | No. cells (median, range) (n=40) |
| --- | --- | --- |
| Live CD8 T cells | 11 (2.6, 17) | 9569 (2576, 16853) |
|  | |  |
|  | % of live CD8^+^ T cells (median, range) | No. cells (median, range) (n=20) |
| IFN-γ Unst | 0.22 (0.054, 0.44) | 22 (3, 41) |
| IFN-γ LL37 | 0.2 (0.05, 0.52) | 23 (2, 40) |
| IFN-γ citLL37 | 0.25 (0.14, 0.47) | 28 (5, 48) |
| IFN-γ TT | 0.24 (0.078, 0.57) | 29 (2, 61) |

|  | % of total (median, range) (n=40) | No. cells (median, range) (n=40) |
| --- | --- | --- |
| Live B cells | 1.7 (0.38, 9.3) | 1620 (383, 9326) |
|  | |  |
|  | % of live B cells (median, range) (n=20) | No. cells (median, range) (n=20) |
| IFN-γ Unst | 0.27 (0.095, 0.56) | 5 (1, 21) |
| IFN-γ LL37 | 0.35 (0, 0.6) | 6 (0, 35) |
| IFN-γ citLL37 | 0.37 (0, 0.57) | 6 (0, 35) |
| IFN-γ TT | 0.32 (0.079, 2.1) | 7 (1, 38) |
